# Proteomics analysis of PK-15 cells infected with porcine parvovirus and the effect of PCBP1 on PPV replication

**DOI:** 10.1128/spectrum.03914-23

**Published:** 2024-05-14

**Authors:** Linqing Wang, Yue Song, Menglong Xu, Chi Zhang, Limeng Zhang, Lu Xia, Zhanyong Wei

**Affiliations:** 1 College of Veterinary Medicine, Henan Agricultural University, Zhengzhou, Henan, China; 2 Molecule Biology Laboratory of Zhengzhou Normal University, Zhengzhou, Henan, China; 3 Ministry of Education Key Laboratory for Animal Pathogens and Biosafety, Zhengzhou, Henan, China; Xinxiang Medical University, Xinxiang, Henan Province, China

**Keywords:** porcine parvovirus, proteomics, PCBP1, replication

## Abstract

Porcine parvovirus (PPV) is one of the most important pathogens that cause reproductive failure in pigs. However, the pathogenesis of PPV infection remains unclear. Proteomics is a powerful tool to understand the interaction between virus and host cells. In the present study, we analyzed the proteomics of PPV-infected PK-15 cells. A total of 32 and 345 proteins were differentially expressed at the early and replication stages, respectively. Subsequent gene ontology annotation and Kyoto Encyclopedia of Genes and Genomes enrichment analysis showed these differentially expressed proteins were significantly enriched in pathways including toll-like receptor signaling pathway, tumor necrosis factor signaling pathway, and viral carcinogenesis. The expression of poly (rC) binding protein 1 (PCBP1) was observed to decrease after PPV infection. Overexpressed or silenced PCBP1 expression inhibited or promoted PPV infection. Our studies established a foundation for further exploration of the multiplication mechanism of PPV.

**IMPORTANCE:**

Porcine parvovirus (PPV) is a cause of reproductive failure in the swine industry. Our knowledge of PPV remains limited, and there is no effective treatment for PPV infection. Proteomics of PPV-infected PK-15 cells was conducted to identify differentially expressed proteins at 6 hours post-infection (hpi) and 36 hpi. Gene ontology and Kyoto Encyclopedia of Genes and Genomes enrichment analysis showed that various pathways participate in PPV infection. Poly (rC) binding protein 1 was confirmed to inhibit PPV replication, which provided potential targets for anti-PPV infection. Our findings improve the understanding of PPV infection and pave the way for future research in this area.

## INTRODUCTION

Porcine parvovirus (PPV) is a non-enveloped DNA virus that belongs to the *Parvovirus* genus in *Parvovirinae* subfamily of *Parvoviridae* family (1). The PPV genome is only about 5,000 bp in length, which contains two gene cassettes (2). The left open reading frame (ORF) encodes the non-structural proteins (NS), while the right ORF encodes the structural or capsid proteins. NS1 gene exhibits high mutation rates, and PPVs are classified into eight types according to NS1 (3–5). Different PPV strains in pigs show varying virulence, and the pathogenic mechanisms are still unclear (6). Studies on the virus-host interaction will help us better understand the mechanism of PPV infection and provide novel targets in combating viral infection.

Isobaric tags for relative and absolute quantitation and tandem mass tag (TMT) are the most widely used protein-labeling methods to identify and quantify protein alterations. Proteomics has been widely used to investigate virus-host interactions in recent years. The outbreak of emerging and re-emerging infectious diseases poses a threat to human health, and proteomics is an indispensable tool for studying the biological characteristics and pathogenesis of pathogens (7). The pandemic of severe acute respiratory syndrome coronavirus 2 (SARS-CoV-2) is a disaster for human society. More than 300 host proteins were identified during SARS-CoV-2 infection (8). These proteins were enriched in spliceosome, proteostasis, and nucleotide biosynthesis pathways, which had the potential to be therapeutic interventions (9). What’s more, 69 compounds that efficiently inhibited SARS-CoV-2 replication *in vitro* were even approved by the Food and Drug Administration or in sub-clinical stage (10). Thus, a proteomic analysis of PPV-infected PK-15 cells was needed to deeply understand the mechanisms of PPV infection.

In this study, TMT-based proteomics technology was used to identify differentially expressed proteins (DEPs) after PPV infection. A total of 4,769 proteins were identified in this program, and proteins that significantly changed were identified. Poly (rC) binding protein 1 (PCBP1) was confirmed to inhibit PPV replication, while the mechanism should be further studied. These results advance our understanding of PPV infection, which provided potential targets for therapeutic strategies.

## MATERIALS AND METHODS

### Cells, viruses, and antibodies

PK-15 cells were cultured in Dulbecco’s modified eagle’s medium (DMEM, Gibco, USA) supplemented with 10% fetal bovine serum (FBS, Gibco, USA) at 37°C with 5% CO_2_. The PPV1 HN-4 strain was isolated from an embryonic death piglet in Henan, China. Monoclonal antibodies against PCBP1 (14523-1-AP) and β-actin (66009-1-Ig) were purchased from Proteintech (China).

### Virus growth curve

The viral growth curve was analyzed as follows. Briefly, PK-15 cells were seeded into six-well plates and infected with PPV at an multiplicity of infection (MOI) of 0.1. Cell pellets were harvested and collected at 3, 6, 12, 24, 36, 48, 72, and 84 hours post-infection (hpi), separately. Cell samples were frozen and thawed three times, and viral DNA was extracted using the TIANamp Virus DNA/RNA Kit (Tiangen, China) following the manufacturer’s instructions. The copy number of virions was determined using quantitative PCR (qPCR).

### RNA extraction and qPCR

Total RNA was extracted from cells using TRIzol Reagent (Takara, Japan) following the manufacturer’s instructions. cDNA was synthesized using a PrimeScript RT Reagent Kit with gDNA Eraser (Takara, Japan). QPCR was performed using Power SYBR Green PCR Master Mix (Vazyme, China). Specific primers are shown in Table 1. β-actin was used as an internal control to normalize gene expression levels. The 2^−ΔΔCt^ method was used to calculate relative expression changes (11).

**TABLE 1:** Primers used in this study

Name	Sequence (5′–3′)	Product size (bp)
PPV VP2	F: ACCGAAGCAACCGCAATTAG	206
	R: CTTTCTAGCTCTTGTGAAGATGTGG	
PCBP1	F: CAGTCTGTCACCGAGTGTGT	83
	R: GTCATGACTCTCCCTTGCGG	
β-actin	F: CTGAACCCCAAAGCCAACCGT	317
	R: TTCTCCTTGATGTCCCGCACG	
PCBP1	F: ATGGATGCCGGTGTGACTG	1,071
	R: CTGCACCCCATGCCCTTC	

### Western blot analysis

PK-15 cells were lysed in radio immunoprecipitation assay lysis buffer (Beyotime, China). The cell extracts were separated by sodium dodecyl sulfate–polyacrylamide gel electrophoresis and transferred to a polyvinylidene difluoride membrane (Millipore, USA). Membranes were blocked with 5% nonfat milk for 2 h at room temperature, and then incubated with the primary antibodies overnight at 4°C. Membranes were washed with tris buffered saline with tween-20 followed by incubation with appropriate horseradish peroxidase conjugated secondary antibodies. Signals were detected using an ECL Chemiluminescence Detection Kit (Millipore, USA).

### Overexpression and RNA interference of PCBP1 in PK-15 cells

The porcine PCBP1 gene was amplified by PCR from porcine liver. The specific primers are shown in Table 1. Then amplified DNA fragment was subcloned into the eukaryotic expression vector pCAGGS-HA and transfected into PK-15 cells using ExFect2000 Transfection Reagent (Vazyme, China) following the manufacturer’s instructions.

Small interfering RNA (siRNA) sequences targeting porcine PCBP1 were designed and synthesized by GenePharma Company (China). PK-15 cells were transfected with siRNA using ExFect2000 Transfection Reagent. The sequence of siRNA345 is 5′-GCGGCUGUAAGAUCAAAGATT-3′.

### TCID_50_ (50% tissue culture infectious dose) assay

PK-15 cells were seeded into 96-well cell culture plates and incubated with serially diluted PPV. Eight replicate wells were conducted at each dilution. Cells were incubated with PPV at 37°C for 1 h, excess virus was removed, and 200 µL of DMEM containing 2% FBS was added to each well. Then the cells were incubated for 3–7 days and monitored daily for cytopathic effect (CPE). The viral titers were calculated using the Reed-Muench method (12).

### Protein sample preparation, trypsin digestion, and TMT labeling

PK-15 cells infected with PPV were collected at 6 hpi and 36 hpi for protein sample preparation. Cells were washed with PBS to remove residual DMEM, harvested with disposable cell scrapers, suspended on ice in 200 µL lysis buffer, and stirred. The samples were further ultrasonicated and boiled. Undissolved cellular debris was removed by centrifugation at 14,000 rpm for 10 min. The supernatants were collected and quantified using a BCA Protein Assay Kit (Dingguo, China).

Protein digestion followed the filter-aided sample preparation procedure described by Wisniewski et al. (13). The resulting peptide mixture was labeled the TMT reagent following the manufacturer’s instructions (Thermo, USA). Briefly, 200 µg proteins for each sample was incorporated into 30 µL STD buffer (4% SDS, 100 mM dithiothreitol, 150 mM Tris-HCl, pH 8.0). The detergent, dithiothreitol, and other low molecular weight components were removed using urea (UA) buffer (8 M urea, 150 mM Tris-HCl, pH 8.0) through repeated ultrafiltration (Microcon units, 30 kD). Subsequently, 100 µL of 0.05 M iodoacetamide in UA buffer was added to block reduced cysteine residues, and the samples were incubated for 20 min in darkness. The filters were washed with 100 µL UA buffer three times and then 100 µL dissolution (DS) buffer (50 mM triethylammonium bicarbonate at pH 8.5) twice. Finally, protein suspensions were digested with 2 µg trypsin (Promega, USA) in 40 µL DS buffer overnight at 37°C, and the resulting peptides were collected as filtrates. The peptide content was estimated by UV light spectral density at 280 nm using an extinction coefficient calculated based on tryptophan and tyrosine frequency in vertebrate proteins. For labeling, each TMT reagent was dissolved in 70 µL of ethanol and added to the respective peptide mixture. Three independent biological replicates were conducted for all experiments.

### Peptide fractionation with strong cation exchange chromatography

TMT-labeled peptides were fractionated by strong cation exchange chromatography using an AKTA Purifier (GE, USA). The dried peptide mixture was reconstituted and acidified with 2 mL buffer A (10 mM KH_2_PO_4_ in 25% acetonitrile, pH 2.7) and loaded onto a Polysulfoethyl 4.6 × 100 mm column (5 µm, 200 Å, PolyLC Inc., USA). The peptides were eluted at a flow rate of 1 mL/min with a gradient of 0%–10% buffer B (500 mM KCl, 10 mM KH_2_PO_4_ in 25% acetonitrile, pH 2.7) for 2 min, 10%–20% buffer B for 25 min, 20%–45% buffer B for 5 min, and 50%–100% buffer B for 5 min. The elution was monitored by absorbance at 214 nm, and fractions were collected every 1 min. The collected fractions (approximately 30 fractions) were finally combined into 10 pools and desalted on C18 cartridges [Empore SPE Cartridges C18 (standard density), bed I.D. 7 mm, volume 3 mL, Sigma, USA]. Each fraction was concentrated by vacuum centrifugation and reconstituted in 40 µL of 0.1% (vol/vol) trifluoroacetic acid. All samples were stored at −80°C until liquid chromatography-electrospray ionization tandem MS (LC-MS/MS) analysis.

### LC-MS/MS analysis

Experiments were performed on a Q Exactive mass spectrometer that was coupled to Easy nLC 1000 (Thermo Fisher Scientific, USA). Ten microliters of each fraction was injected for nanoLC-MS/MS analysis. The peptide mixture (5 µg) was loaded onto a C18-reversed-phase column (15 cm long, 75 µm inner diameter) packed in-house with RP-C18 5 µm resin in buffer A (0.1% formic acid) and separated with a linear gradient of buffer B (80% acetonitrile and 0.1% formic acid) at a flow rate of 250 nL/min controlled by IntelliFlow technology for over 60 min. MS data were obtained using a data-dependent top10 method dynamically selecting the most abundant precursor ions from the survey scan (300–1,800 m/z) for higher-energy collision-induced dissociation (HCD) fragmentation. Determining the target value is based on predictive automatic gain control. Dynamic exclusion duration was 60 s. Survey scans were acquired at a resolution of 70,000 at m/z 200, and the resolution for HCD spectra was set to 17,500 at m/z 200. The normalized collision energy was 30 Ev, and the underfill ratio, which specifies the minimum percentage of the target value likely to be reached at maximum fill time, was defined as 0.1%. The instrument was operated with peptide recognition enabled.

### Sequence database searching and data analysis

MS/MS spectra were searched using the MASCOT engine (Matrix Science, UK; version 2.2) embedded into Proteome Discoverer 1.3 (Thermo Electron, USA) against the Sus_scrofa_110599 uniprot and the decoy database. For protein identification, the following options were used. Peptide mass tolerance was 20 ppm, MS/MS tolerance was 0.1 Da, enzyme was trypsin, missed cleavage was 2, fixed modification: carbamidomethyl (C), TMT 16plex(K), TMT 16plex(N-term), variable modification: oxidation(M), false discovery rate ≤ 0.01.

### Bioinformatics

The gene ontology (GO) annotation and Kyoto Encyclopedia of Genes and Genomes (KEGG) enrichment were performed using the R Cluster profiler package. The volcano plot and heat map were drawn using the R ggplot2 and heat map packages, respectively. Gene set enrichment analysis was performed using Gene Set Enrichment Analysis software (http://www.broadinstitute.org/gsea).

### Statistical analysis

All experiments were performed in triplicate. Data are presented as mean ± standard deviation. *P* values were calculated using Student’s *t*-test or one-way analysis of variance. Differences are defined as *, 0.01 < *P* < 0.05; **, *P* < 0.01.

## RESULTS

### Growth curve of PPV in PK-15 cells

**Fig 1:**
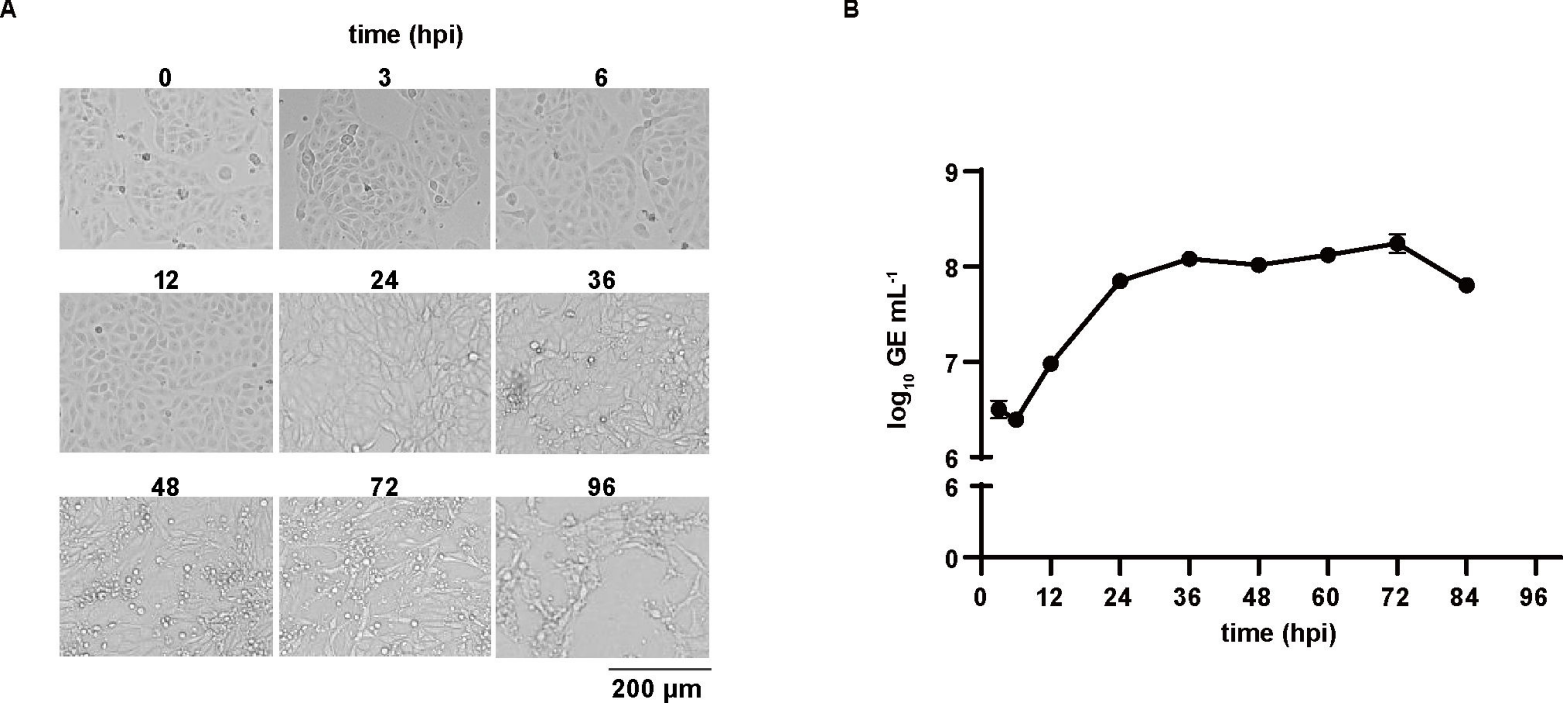
Proliferation of PPV in PK-15 cells. (**A**) PK-15 cells were infected with PPV (MOI = 0.1), then cultured for 96 h. Cell morphology was observed at different time points after PPV infection. Scale bar = 100 µm. (**B**) Growth curve of PPV in PK-15 cells. PK-15 cells were infected with PPV and collected at different time points. The genomic DNA of PPV was detected by qPCR. GE means genome equivalents.

Viral proliferation is crucial to optimize virus inoculation and collection, which offer guidance for proteomics analysis. The growth curve of PPV in PK-15 cells was detected. No obvious CPE was observed at 12 hpi. The cell boundaries became blurred at 24 hpi, clustered into a group at 36 hpi, and gradually shed at 48 hpi (Fig. 1A). The growth curve showed PPV rapidly replicated from 6 hpi to 24 hpi, peaked at 36 hpi, stabilized at 48–72 hpi, and began to decline at 84 hpi (Fig. 1B). Accordingly, 6 hpi and 36 hpi were considered to be the early and replication stages of PPV infection, respectively.

### Differential protein analysis after PPV infection

**Fig 2:**
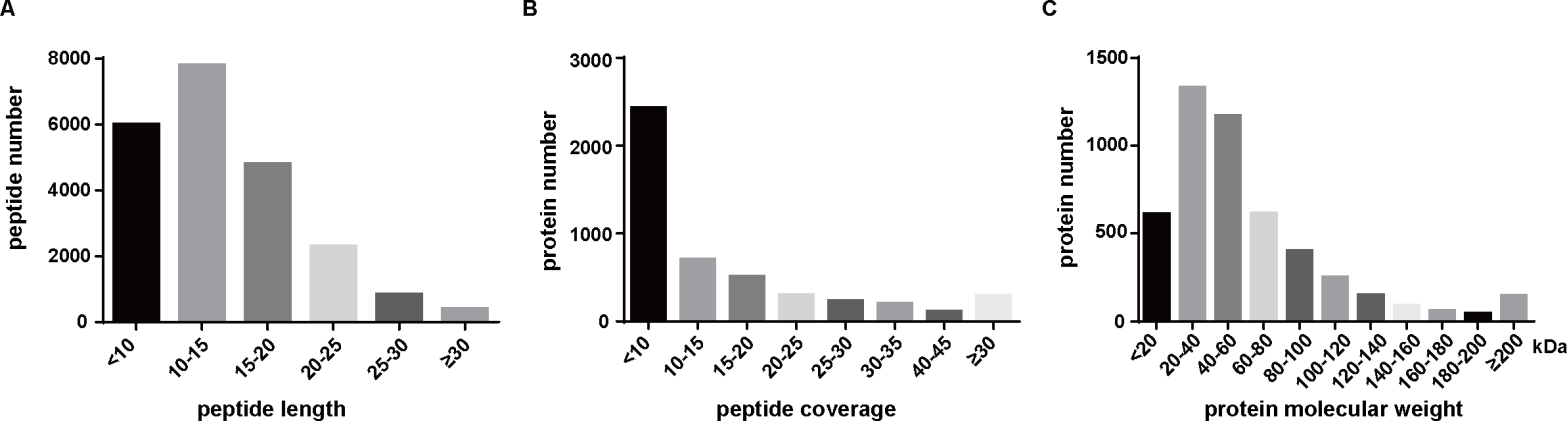
The quantification of peptides identified by mass spectrometry. (**A**) Peptide length distribution. (**B**) Distribution of proteins based on their number of identified peptides. (**C**) Distribution of these proteins based on their molecular weight.

After TMT proteomics, the database (uniprot_Sus_scrofa_110599) was used to analyze the identified mass spectra. A total of 168,686 secondary mass spectra were obtained, 44,634 peptides were identified, and 21,100 specific peptides were matched. Among these specific peptides, 4,769 proteins were identified. The distribution of peptide length, peptide coverage, and protein molecular weight is shown in Fig. 2. Only protein fold changes 1.5 or 0.67, and *P* value <0.05 were considered to be significantly different expression proteins. The red dots represent significantly upregulated proteins, the blue dots represent significantly downregulated proteins, and the gray dots are proteins with no significant difference.

**Fig 3:**
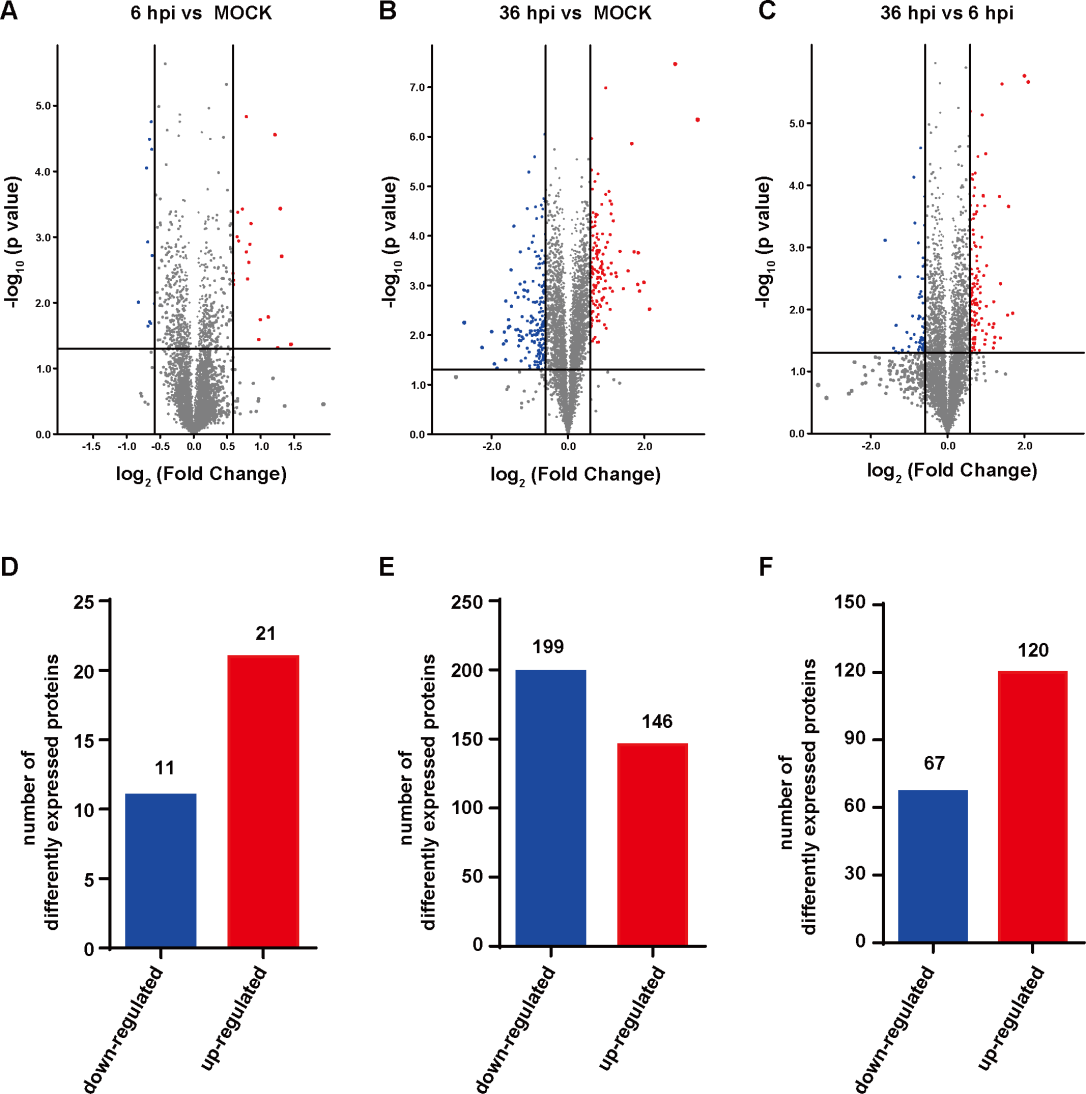
DPEs identified in this study. (A–C) Volcano plot of DPEs. Each point represents a detected protein. Red dots represent upregulated proteins, blue dots represent downregulated proteins, and the gray dots indicate the non-significant differentially expressed proteins. (D–F) Quantitative statistics of upregulated and downregulated proteins at 6 hpi (**D**), 36 hpi (**E**), and 36 hpi vs 6 hpi (**F**).

**Fig 4:**
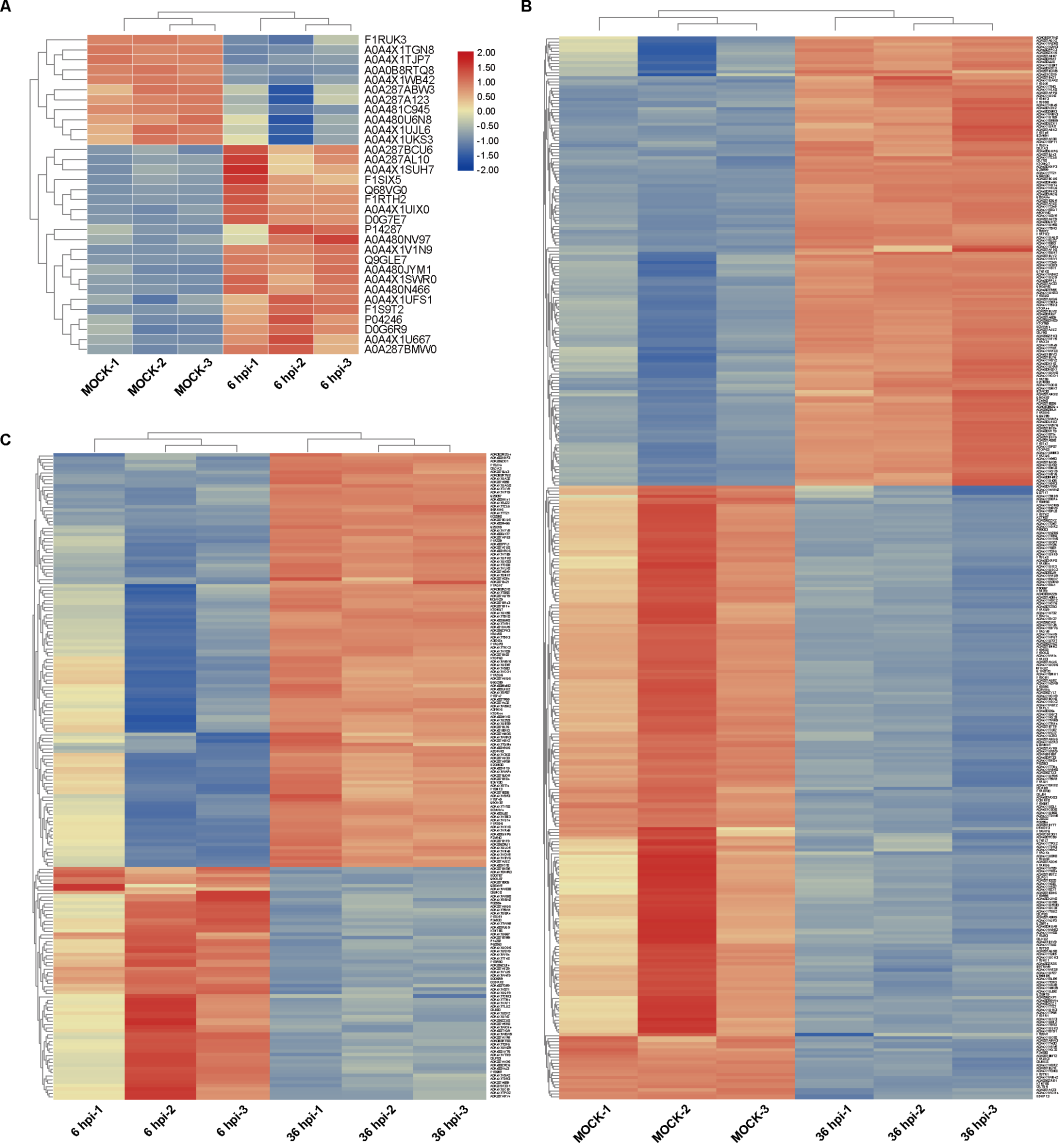
Hierarchical cluster analysis of DEPs among different groups. (**A**) DEPs at 6 hpi group. (**B**) DEPs at 36 hpi group. (**C**) DEPs between 36 hpi group vs 6 hpi group.

Relative to the control cells, 21 proteins were expressed at high levels, and 11 proteins were expressed at lower levels at 6 hpi (Fig. 3A and D). 345 DEPs were detected at 36 hpi compared to the mock group, among which 146 were significantly upregulated and 199 significantly downregulated (Fig. 3B and E). And a total of 187 DEPs were screened when comparing DEPs at 36 hpi to 6 hpi (Fig. 3C and F). To provide a visualization of overall protein changes, the DEPs of each group were analyzed in the form of heat maps and hierarchical cluster analysis. Gene Cluster 3.0 software was used for cluster analysis, and the results are shown in Fig. 4.

### GO and KEGG enrichment analysis

**Fig 5:**
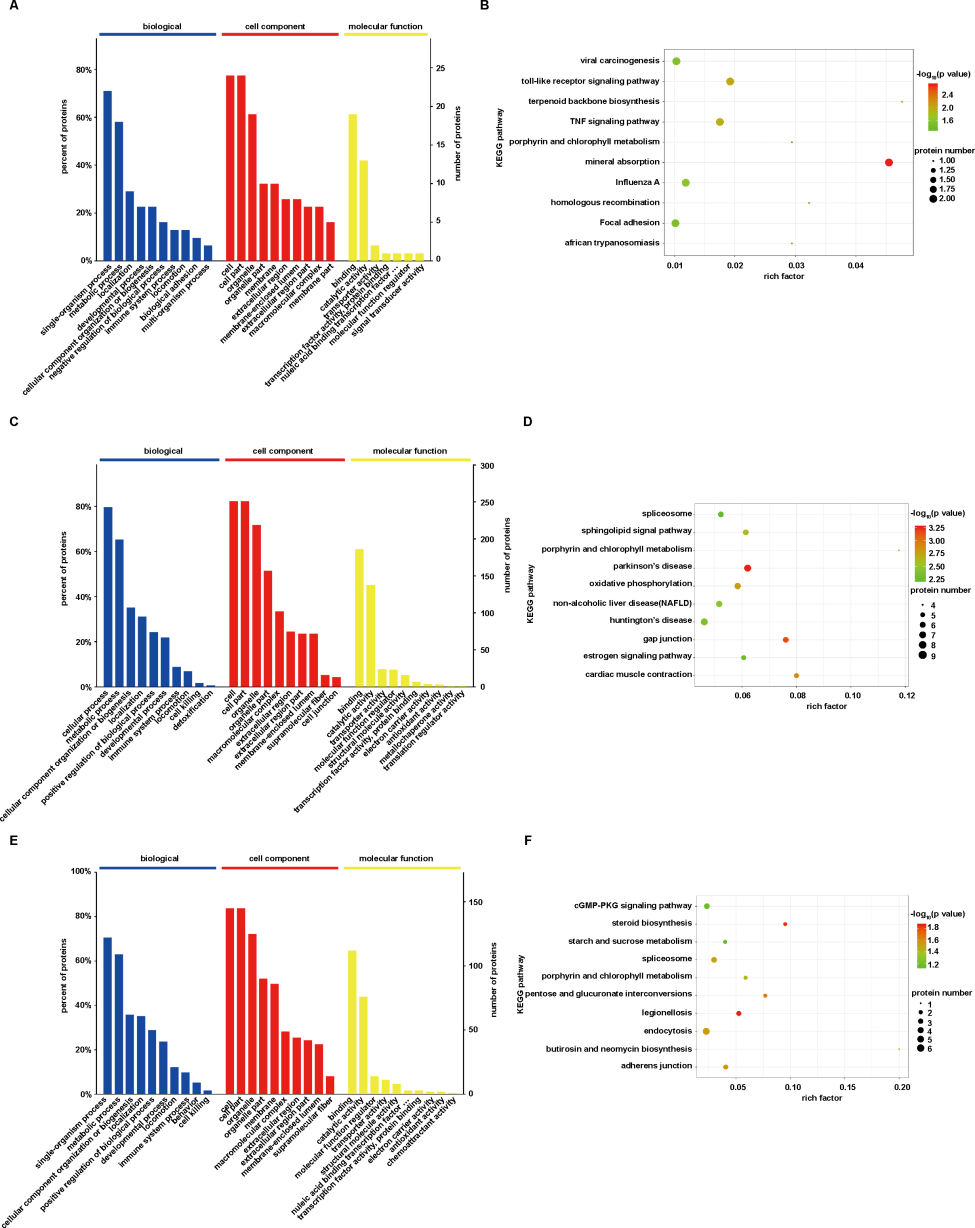
Enrichment of GO terms and KEGG pathway of DEPs. (**A**) GO enrichment analysis of DEPs at 6 hpi group. (**B**) KEGG enrichment analysis at 6 hpi group. (**C**) GO enrichment analysis at 36 hpi group. (**D**) KEGG enrichment analysis at 36 hpi group. (**E**) GO enrichment analysis between 36 hpi vs 6 hpi. (**F**) KEGG enrichment analysis between 36 hpi vs 6 hpi.

To classify the DEPs, GO and KEGG enrichment analyses were conducted. The most significant enriched GO terms and KEGG pathways were performed by Fisher’s exact test (*P* < 0.05). The biological process (BP), cellular component (CC), and molecular function (MF) were involved in the submitted proteins. Top 10 biological processes are shown in Fig. 5.

GO and KEGG pathway enrichment analyses of 6 hpi group are depicted in Fig. 5A and B. BP showed that DEPs were predominantly enriched in single-organism processes, metabolic processes, localization, and developmental processes. CC revealed that DEPs were primarily enriched in cells and organelles, such as membranes and extracellular regions. MF indicated that DEPs were enriched in binding, catalytic activity, transporter activity, and more. A total of 52 signaling pathways were enriched in KEGG analysis, primarily in mineral absorption, toll-like receptor signaling pathway, tumor necrosis factor signaling pathway, and viral carcinogenesis.

At 36 hpi, the DEPs were dominantly enriched in cellular process, metabolic process, cellular component organization, or biogenesis in BP (Fig. 5C). In the KEGG enrichment analysis,196 signaling pathways were enriched, including gap junction, oxidative phosphorylation, sphingolipid signaling pathway, and so on (Fig. 5D). We also analyzed DEPs between 36 hpi vs 6 hpi. GO enrichment terms and KEGG enrichment analysis are shown in Fig. 5E and F.

### PPV inhibits PCBP1 expression in PK-15 cells

**Fig 6:**
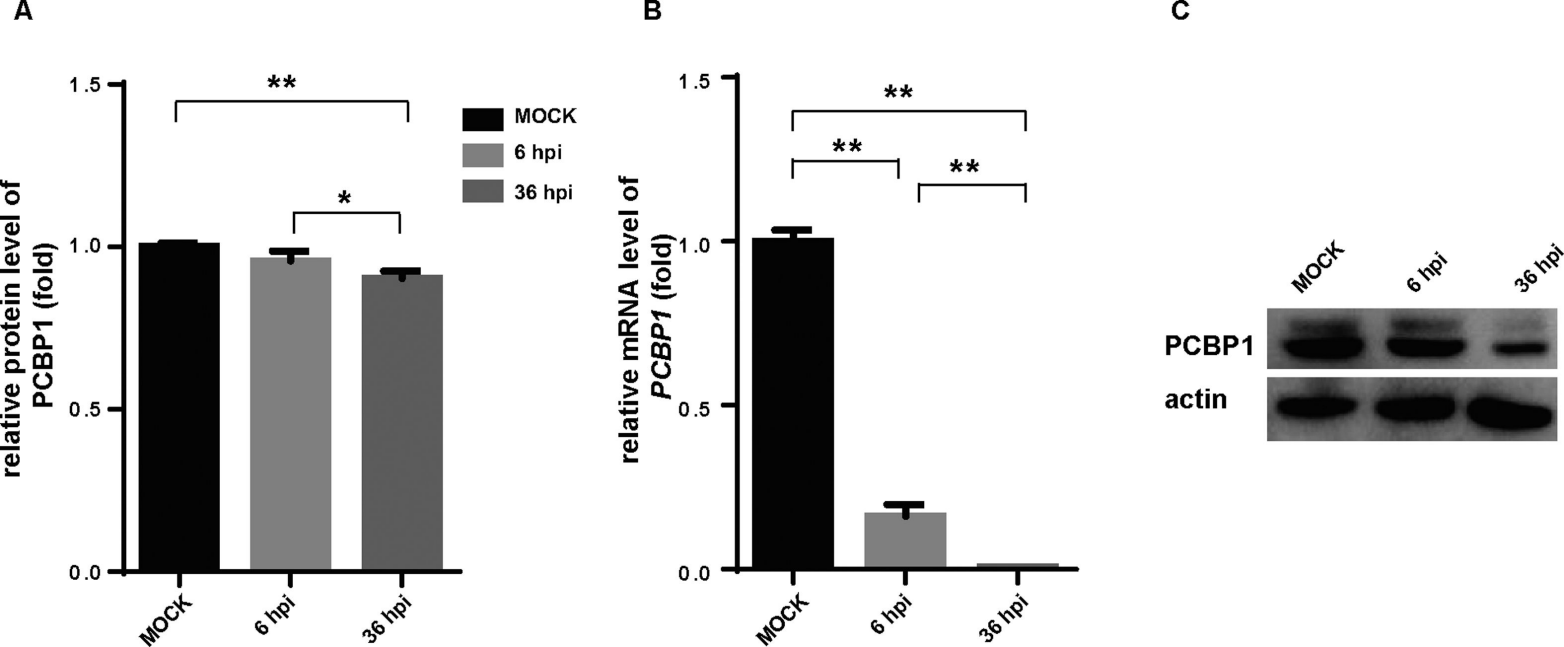
PPV inhibits PCBP1 expression. (**A**) The relative expression of PCBP1 post-PPV infection from the TMT proteome results. (**B**) qPCR analysis of PCBP1 mRNA level. (**C**) The expression of PCBP1 was analyzed by WB. *P < 0.05 and **P < 0.01.

Proteomics data indicated significant variation in PCBP1 expression at 36 hpi (Fig. 6A). PCBP1 expression level was further verified by qPCR and western blot. Results showed that PCBP1 mRNA expression level gradually decreased with prolonged PPV infection (Fig. 6B). Similarly, the expression of PCBP1 was also decreased at 6 hpi and 36 hpi in PK-15 cells (Fig. 6C). These results showed that PCBP1 levels in cells gradually decreased as PPV infection progressed, suggesting PCBP1 may be involved in the PPV life cycle.

### PCBP1 inhibits PPV replication

**Fig 7:**
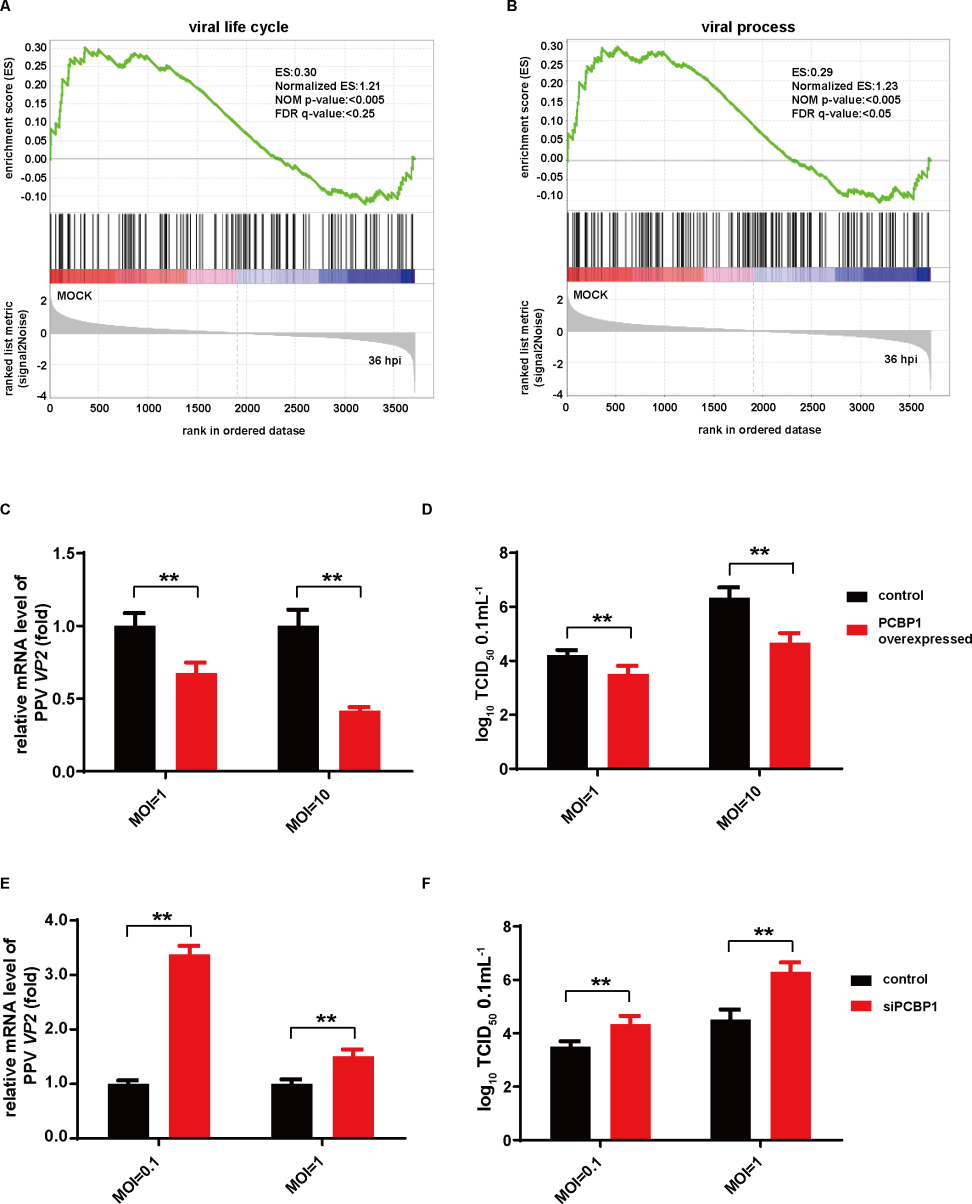
PCBP1 inhibits PPV replication. (**A and B**) GSEA enrichment of PCBP1. (**C and D**) PCBP1 overexpression inhibits PPV infection. PCBP1 was overexpressed and then infected with PPV at different MOIs. PPV VP2 level was determined with qPCR (**C**), and viral titers were determined by TCID_50_ assay (**D**). (**E and F**) Knockdown of PCBP1 enhances PPV infection. PCBP1 was silenced and infected with PPV in PK-15 cells. Levels of PPV VP2 were determined with qPCR (**E**), and viral titers of PPV were determined with TCID_50_ (**F**). *P < 0.05 and **P < 0.01.

To analyze the precise role of PCBP1 during PPV infection, GO terms containing PCBP1 were selected for Gene Set Enrichment Analysis (GSEA, http://software.broadinstitute.org/gsea/index.jsp). The results showed that two gene sets, viral life cycle (Fig. 7A) and viral process (Fig. 7B), were significantly enriched, indicating PCBP1 was closely related to the PPV life process.

To assess the influence of PCBP1 on PPV replication, PCBP1 was overexpressed or silenced in PK-15 cells. Cells were transfected with pCAGGS-HA vector or plasmids encoding PCBP1 for 24 h and infected with PPV for another 24 h. As shown in Fig. 7C, lower PPV VP2 levels were observed in PCBP1 overexpressed cells compared to control cells. Additionally, PCBP1 inhibited the viral titer of PPV (Fig. 7D). To further confirm the effect of PCBP1 on PPV infection, PCBP1 was knockdown. As shown in Fig. 7E and F, viral replication was enhanced in siPCBP1-treated cells. In summary, these results suggested that PCBP1 inhibits PPV replication.

## DISCUSSION

PPV is an important agent causing reproductive failure in swine and has led to huge economic losses in the swine industry (14). Over the past two decades, several novel strains of PPV have been identified in pigs (15, 16), which makes it more difficult to prevent and control PPV infection. It is necessary to study PPV-cell interaction at the protein level. Numerous DEPs that provided a whole view of PPV infection were identified from proteomics. Through classification and diverse functional enrichment analysis, we found these DEPs have different subcellular localizations and are involved in various physiological processes, which pointed out the further research direction of PPV infection.

The growth curve of PPV showed a lower growth rate at 6 h, which was selected to represent the early stage of PPV infection. GO analysis highlighted that cellular components were primarily concentrated in cells, cell parts, and organelles. Molecular function was mainly focused on binding, catalytic activity, and transport activity. These proteins may be involved in the early physiological processes of PPV infection. Among these DEPs, calponin-homology domain-containing protein (CCDC88A) was significantly upregulated. CCDC88A was enriched in cytoskeleton composition. The cytoskeleton plays an important role in maintaining normal cell physiological state and vesicular trafficking (17, 18). Research shows that cytoskeletons are exploited by viruses to promote multiple steps of the infection cycle, including viral entry, genome replication, and intracellular trafficking of viral components (19). Oliva et al*.* found that SARS-CoV-2 takes advantage of the host transport machinery that functions through the cytoskeleton for their active movement into the cell (20). Virus nucleoprotein complexes utilize cytoskeletal to travel long distances in the cytoplasm from the cell surface to the replication and transcription sites of viral DNA and induce infection (21). Another upregulated DEP was karyopherin subunit alpha 2 (KPNA2), a nuclear transport protein that transports cargo proteins into the nucleus (22). These altered proteins indicated that PPV utilizes cytoskeleton to enter the cell and transport viral components to appropriate organelle at the early stages of PPV infection.

The number of DEPs at the late infection stage was notably higher compared to that in the early stage. A total of 196 signaling pathways were enriched in the PPV replication stage (36 hpi). The gap junction pathway was significantly enriched, with seven DEPs involved in material exchange between cells (23), suggesting an impact on intercellular information exchange during PPV infection. Lipase A, associated with the steroid biosynthesis pathway, was increased at 36 hpi. Increased lipase A expression leads to sterol and lipid release, and lipids often act as co-receptors or contribute to viral replication complex formation. Glycophospholipid globulin has been demonstrated to be the receptor of human parvovirus B19 entry into cells (24); human immunodeficiency virus chooses lipid raft microregions-rich region on the plasma membrane to release the virus outside the cell (25). Some viruses even regulate cell lipid metabolism to promote virus proliferation (26). These suggested that lipid metabolism may play a vital role in PPV infection.

Among the DEPs, PCBP1 attracted our interest. PCBP1 participates in several virus-related signaling pathways, and its expression is closely related to viral replication. PCBP1 participates in antiviral innate immune regulation through STAT3-mediated nuclear factor kappa B (NF-κB) activity inhibition (27, 28), cyclic guanosine monophosphate-adenosine monophosphate (GMP-AMP) synthase protein (29), and mitochondrial antiviral signaling protein (MAVS) pathways (30). PCBP1 is highly expressed in various tissues and organs (31) and participates in mRNA shuttle, transcription, and translation (32). Proteomic analysis showed a gradual decrease in PCBP1 expression after PPV infection, and considerable inhibition of PCBP1 on PPV infection was demonstrated. However, the molecular mechanism of PCBP1 anti-PPV infection remains unclear. Further research is essential to address these queries and provide valuable insights in the development of antiviral strategies.

### Conclusions

Overall, our study presents new perspectives on PPV infection. We investigate the viral pathogenesis and host responses to PPV infection by proteomics, which pave the way for more efficient strategies to control and prevent PPV infection in the swine industry.

## Data Availability

Data are available via ProteomeXchange with identifier PXD050764.
